# MicroRNA-101a regulates microglial morphology and inflammation

**DOI:** 10.1186/s12974-017-0884-8

**Published:** 2017-05-30

**Authors:** Reiko Saika, Hiroshi Sakuma, Daisuke Noto, Shuhei Yamaguchi, Takashi Yamamura, Sachiko Miyake

**Affiliations:** 1 Department of Immunology, National Institute of Neuroscience, National Center of Neurology and Psychiatry, 4-1-1, Ogawahigashi-cho, Kodaira, Tokyo, Japan; 2 Department of Neurology, Shimane University Faculty of Medicine, 89-1 Enya-cho, Izumo, Shimane Japan; 3 Department of Brain Development and Neural Regeneration, Tokyo Metropolitan Institute of Medical Science, 2-1-6 Kamikitazawa, Setagaya, Tokyo, Japan; 4 Department of Immunology, Juntendo University School of Medicine, 2-1-1 Hongou, Bunkyo, Tokyo, Japan

**Keywords:** Microglia, Differentiation, Hematopoietic cell, microRNA-101a, Cytokine

## Abstract

**Background:**

Microglia, as well as other tissue-resident macrophages, arise from yolk sac progenitors. Thus, it is likely that the central nervous system environment is critical for the acquisition of a distinct microglial phenotype. Several microRNAs that are enriched in the brain play crucial roles in brain development and may also play a role in the differentiation of microglia.

**Methods:**

To track the differentiation of hematopoietic cells into microglia, lineage-negative bone marrow cells were co-cultured with astrocytes in the absence or presence of microRNAs or their inhibitors. Microglia-like cells were identified as small, round cells that were immunopositive for CD11b, Iba1, CX3CR1, and triggering receptor expressed on myeloid cells (TREM)-2.

**Results:**

Five microRNAs (miR-101a, miR-139-3p, miR-214^*^, miR-218, and miR-1186) were identified as modifiers of the differentiation of bone marrow-derived microglia-like cells. Among them, miR-101a facilitated the differentiation of bone marrow cells into microglia-like cells most potently. Small, round cells expressing CD11b, Iba1, CX3CR1, and TREM-2 were predominant in cells treated by miR-101a. miR-101a was abundantly expressed in non-microglial brain cells. Transfection of miR-101a into microglia significantly increased the production of IL-6 in response to LPS. Finally, miR-101a downregulated the expression of MAPK phosphatase-1.

**Conclusions:**

miR-101a, which is enriched in the brain, promotes the differentiation of bone marrow cells into microglia-like cells.

**Electronic supplementary material:**

The online version of this article (doi:10.1186/s12974-017-0884-8) contains supplementary material, which is available to authorized users.

## Background

MicroRNAs (miRNAs) are small non-coding RNAs that function as guide molecules in RNA silencing [1]. miRNAs are also enriched in the central nervous system (CNS) and play crucial roles in the development and plasticity of the brain [2]. Certain miRNAs regulate differentiation, activation, and polarization of microglia [3]. miR-124, one of the most abundant miRNAs in neurons, is expressed in microglia and promotes microglial quiescence by inhibiting the C/EBP-α-PU.1 pathway [4]. miR-155 is elevated in M1-polarized microglia and regulates their pro-inflammatory responses [5]. Recently, miR-9 was reported to promote microglial activation by targeting MCPIP1 [6].

Microglia arise from primitive yolk sac macrophages and develop independently of the hematopoietic system [7]. Furthermore, a recent study demonstrated that other tissue-resident macrophages also originate from yolk sac progenitors [8]. This evidence suggests that the developmental fate of tissue macrophages is influenced by the environment in which cells are placed. In addition to colony-stimulating factors including interleukin-34, which dictate the developmental fate of microglia [9], miRNAs enriched in the CNS may also play a role in the acquisition of a distinct microglial phenotype.

A murine bone marrow chimera model suggests that hematopoietic cells have the potential to develop into microglia [10]. We developed an in vitro model in which lineage-negative bone marrow cells co-cultured with astrocytes differentiated into microglia-like cells [11, 12]. In this model, bone marrow-derived cells showed two morphological forms, namely, small round (SR) cells having relatively small, round-shape soma or large flat (LF) cells having polymorphic soma and pseudopodia. Only SR cells expressed triggering receptor expressing on myeloid cells-2 (TREM-2) that is predominantly expressed on microglia [13]. This indicated that SR cells had phenotypical similarity to microglia and thus we defined these cells as microglia-like cells [12].

In the present study, we sought to identify miRNAs that affect the phenotype of microglia using an in vitro co-culture model and immortalized microglial cell line. We show that miR-101a modulates microglial morphology and inflammation.

## Methods

### Mice

Six-week-old female C57BL/6J (B6) mice were purchased from the Japan CLEA Laboratory Animal Corporation (Tokyo, Japan). Beta-actin promoter-driven enhanced green fluorescence protein (GFP) transgenic mice on a B6 background were kindly provided by Dr. Masaru Okabe (Osaka University, Japan).

### Isolation of murine LN^−^ cells

To isolate lineage negative (LN^−^) cells, bone marrow cells were collected from B6 mice or GFP mice by flushing the femora and tibiae of the hind limbs with phosphate-buffered saline. Erythrocytes were lysed using ammonium chloride-potassium buffer. LN^−^ cells were negatively selected using antibodies (Abs) against lineage-specific markers (CD3, CD4, CD5, CD8α, CD11b/MAC-1α, B220, Gr-1, and TER-119; R&D Systems, Minneapolis, MN, USA) and immunomagnetic beads (Dynal, Oslo, Norway).

### Isolation of microglia from the CNS

To isolate microglia from the CNS of adult mice, we dissociated the brain tissues of 6-week-old mice using Neural Tissue Dissociation Kits (P) (Miltenyi Biotec, Bergisch-Gladbach, Germany). Mononuclear cells were obtained through a density gradient from the interface between 27 and 72% Percoll (GE Healthcare, Waukesha, WI, USA) layers. Microglia were isolated from the single-cell suspension by MACS Technology using CD11b (Microglia) Microbeads (Miltenyi Biotec). The purity of microglia was >98%.

### Isolation of peritoneal macrophages and bone marrow-derived macrophages

To isolate peritoneal macrophages, peritoneal lavage fluid was collected from B6 mice by washing the peritoneum with 5 mL Hank’s balanced salt solution (HBSS; Gibco, Carlsbad, CA, USA). Cells were cultured overnight and adherent cells were harvested as macrophages. To obtain bone marrow-derived macrophages, bone marrow cells were isolated from the femur and the tibia of B6 mice and cultured for a week in the presence of recombinant murine M-CSF (20 ng/mL, BioLegend, San Diego, CA).

### Induction of microglia-like cells

Primary mixed glial cell cultures were prepared from the brains of postnatal 3–5-day-old (P3–P5) B6 mice, as previously described [12]. Astrocytes were prepared after removal of CD11b^+^ cells using Dynabeads (Dynal) conjugated with anti-CD11b Ab. The purity of astrocytes was >96% as determined by anti-GFAP and anti-CD11b immunofluorescence. Astrocytes were then seeded into culture flasks, 96-well plates, or Lab-Tek II 8-well chamber slides (Thermo Fisher Scientific, Waltham, MA, USA) at a density of 7.5 × 10^6^ cells per flask, 2.5 × 10^4^ cells per 96-well, or 1.0 × 10^5^ cells per chamber slide well and cultured in the medium described above for 5 days to form a confluent monolayer. LN^−^ cells were seeded on astrocytes at a density of 1.5 × 10^6^ cells per flask or 0.5 × 10^4^ cells per 96-well and chamber slide well and cultured for 7 days.

### Culture of MG6 cells

A murine microglia cell line of MG6 cells was kindly provided by Dr. Hiroshi Kitani (National Institute of Agrobiological Sciences, Japan) [14]. We cultured MG6 cells in Dulbecco’s modified Eagle’s medium, supplemented with 10% fetal calf serum (FCS), 1% glucose, 1% l-glutamine, 1% penicillin/streptomycin, 10 μg/ml insulin, and 0.1 mM 2-mercaptoethanol at 37 °C in a humidified atmosphere of 5% CO_2_ and 95% air.

### Screening using microRNA inhibitor library

We screened miRNAs that modify microglial differentiation and activation using the miRCURY LNA^TM^ microRNA inhibitor library (Exiqon, Vedbaek, Denmark). A total of 739 miRNA inhibitors were screened. miRNA inhibitors were suspended in Opti-MEM and HiperFect transfection reagent (Qiagen, Hilden, Germany) and were incubated for 15 min at room temperature. Then, miRNA transfectants were added to LN^−^ cell-astrocyte co-culture seeded on 96-well plates at a final concentration of 40 nM on days 0 and 3. On day 7, wells were observed microscopically by two authors (RS and HS) in a blinded manner and morphological findings were independently assessed in a semi-quantitative manner. Wells were scored as hits when both investigators judged that the number of SR cells changed significantly.

### miRNA mimic and inhibitor treatment

For immunohistochemistry, LN^−^ cell-astrocyte co-culture seeded on 8-well chamber slides were transfected with miRNA inhibitors and mimics using the same protocol as described above. For ELISA, MG6 cells were seeded at a density of 2 × 10^4^ cells per 96-well. The next day, miRNA inhibitors and mimics were transfected at a final concentration of 40 nM with Lipofectamine RNAiMAX (Thermo Fisher Scientific).

### Immunohistochemistry

LN^−^ cells that were isolated from GFP mice and cultured on astrocytes were fixed in 4% paraformaldehyde. After treatment with protein block (Dako, Denmark), samples were immunostained with: (1) biotin-conjugated anti-CD11b Ab (BioLegend, San Diego, CA, USA) followed by DyLight 649-Streptavidin (Jackson ImmunoResearch, West Grove, PA, USA), (2) anti-TREM2 Ab (R&D systems) followed by rhodamine-anti-sheep IgG (Jackson ImmunoResearch), (3) anti-CX3CR1 (R&D systems) followed by Cy5-anti-goat IgG (Jackson ImmunoResearch), or (4) anti-Iba1 (Wako Pure Chemical, Osaka, Japan) followed by rhodamine-anti-rabbit IgG Ab (Jackson ImmunoResearch). Images were acquired using an FV1000-D microscope (Olympus, Tokyo, Japan). To quantify the number of cells, ten fields under a ×20 objective were randomly selected, and GFP-positive or immunostained cells were counted using ImageJ software (National Institutes of Health, Bethesda, MD, USA). SR cells and LF cells were defined as cells with a circularity of >0.8 and ≤0.8, respectively.

### Enzyme-linked immunosorbent assay (ELISA)

After stimulation with LPS, we collected the cell supernatants and analyzed them using the BD OptEIA^TM^ Set (BD Biosciences, Franklin Lakes, NJ, USA) for mouse IL-10, TNFα, and IL-6 and the Ready-SET-Go! kit (eBioscience, San Diego, CA, USA) for mouse IL-1β.

### Real-time PCR analysis

We isolated mRNA from MG6 and brain cells using the RNeasy Mini Kit (Qiagen), and we isolated miRNA using the miRNeasy Mini Kit (Qiagen). We performed reverse transcription for mRNA using PrimeScript RT Master Mix (Perfect Real Time) (Takara Bio, Otsu, Japan) and real-time PCR with SYBR Premix Ex Taq^TM^ II (Tli RNaseH Plus) (Takara Bio). For miRNA expression analysis of miR-101 and snoRNA202, we used TaqMan MicroRNA assays (Applied Biosystems, San Francisco, CA, USA).

### MTT assay

Viability of MG6 cells was assessed using the CellTiter 96^®^ Aqueous One Solution Cell Proliferation Assay kit (Promega, Madison, WI, USA). MG6 cells (2.5 × 10^4^ cells per well) were seeded on 96-well plate and cultured for 24 h. CellTiter 96^®^ Aqueous One Solution Reagent (20 μL per well) were added to each well and plate was incubated at 37 °C for 1 h. The quantity of formazan product that is proportional to the number of living cells in culture was measured by absorbance at 490 nm.

### Statistical analysis

Results represent at least three independent experiments. Data are presented as mean ± S.D. Statistical significance was determined by one-factor ANOVA, Kruskal-Wallis test, Student’s *t* test, and Mann-Whitney test.

## Results

### Identification of miR-101a as a modulator of microglial morphology by miRNA inhibitor library screening

We speculated that secreted factors including miRNAs induced the differentiation of microglia-like cells. In order to screen miRNAs that affect microglial development, GFP^+^LN^−^ cells were co-cultured with astrocytes and then were treated with an miRNA inhibitor library. We selected this co-culture model instead of primary microglia because it has been reported that primary macrophage is difficult to transfect. Among 739 miRNA inhibitors, 38 showed cytotoxic effect on co-culture and were excluded from the analysis. There were 27 hits among 701 inhibitors: 22 inhibitors increased the number of SR cells and 5 inhibitors decreased them. We thereafter focused on five miRNA inhibitors (miR-101a, miR-139-3p, miR-214^*^, miR-218, and miR-1186) that decreased the number of SR cells.

**Fig. 1:**
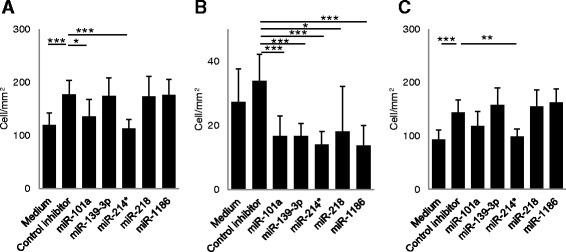
LN^−^ cells derived from GFP mice co-cultured with astrocytes in the presence of miRNA inhibitors. **a** The numbers of total GFP^+^ cells, **b** GFP^+^ small, round cells, **c** and GFP^+^ large, flat cells in the presence of each miRNA inhibitor are shown (*n* = 8). Data are presented as means ± S.D. *P < 0.05, **P < 0.01, ***P < 0.001 by Kruskal–Wallis test followed by Steel–Dwass multiple comparison

Control miRNA inhibitor significantly increased the number of total cells (Fig. 1a). Inhibitors of miR-101a and miR-214^*^ decreased the number of total cells in culture compared to those treated with control inhibitor, but difference was not significant when compared to untreated cells (Fig. 1a). All five miRNA inhibitors decreased the number of SR cells (Fig. 1b). Control miRNA inhibitor significantly increased the number of LF cells while miR-214^*^ inhibitor decreased the number of LF cells compared to control inhibitor (Fig. 1c).

**Fig. 2:**
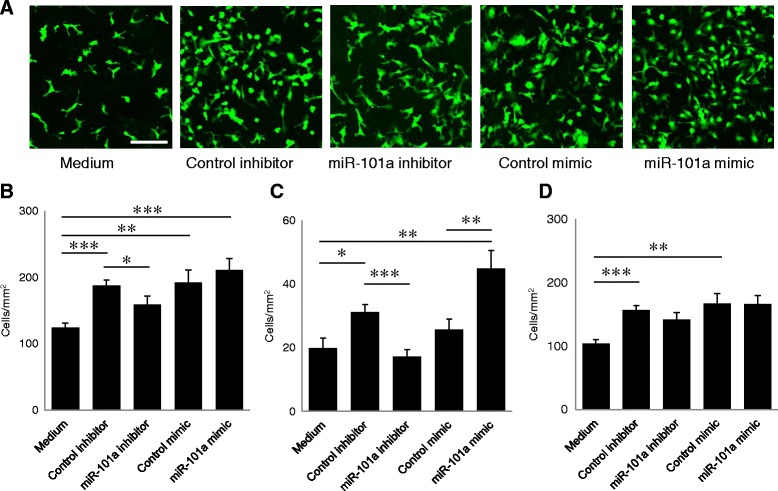
GFP^+^LN^−^ cells co-cultured with astrocytes in the presence of miRNA-101a inhibitor or mimic. **a** Fluorescent microscopic images of GFP^+^LN^−^ cells cultured with miR-101a inhibitors or mimics. *Bar* = 100 μm. **b** Numbers of total GFP^+^ cells, **c** GFP^+^ small, round cells, **d** and GFP^+^ large, flat cells are shown (*n* = 20). Data are presented as means ± S.D. *P < 0.05, **P < 0.01, ***P < 0.001 by Mann-Whitney test

Since it was expected that miRNA mimics would have the opposite effect of miRNA inhibitors, we assessed the effect of five miRNA mimics using the same co-culture system and found that miR-101a was the only miRNA whose inhibitor and mimic exerted opposite effects. As shown in Fig. 2a, both the inhibitor and mimic of miR-101 altered the proportion of SR cells. Similarly to Fig. 1, when compared to medium, control inhibitor significantly increased the number of total, SR, and LF cells, and control mimic also increased total and LF cells (Fig. 2a–d). Since we speculated that these were due to the non-sequence-specific immune activation caused by transfection of miRNA, the effects of miR-101a inhibitor/mimic were compared with those of control inhibitor/mimic thereafter. The miR-101a inhibitor decreased the number of total cells as well as the number of SR cells compared to control inhibitor, while the miR-101a mimic increased the number of SR cells compared to both medium and control mimic (Fig. 2b, c). Both the inhibitor and mimic of miR-101a did not affect the number of LF cells (Fig. 2d). We confirmed that exogenous miRNAs were incorporated into the cells (Additional file 1: Figure S1). These results demonstrate that miR-101a facilitates the differentiation of LN^−^ cells into SR cells.

### miR-101a facilitates induction of TREM-2-positive microglia-like cells from bone marrow LN^−^ cells

**Fig. 3:**
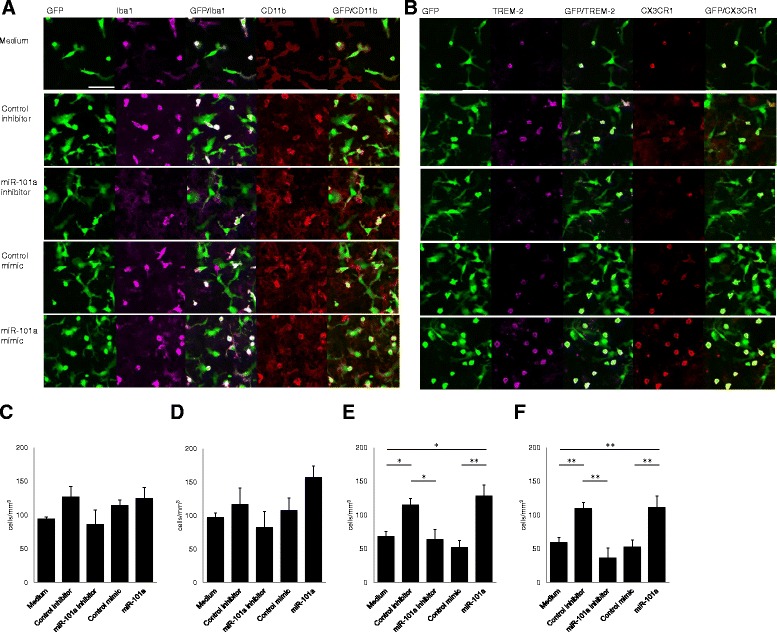
Immunocytochemistry of GFP^+^LN^−^ cells in the presence of miR-101a inhibitor or mimic. **a** Cultures were stained with antibodies against Iba1 and CD11b. **b** Cultures were stained with antibodies against TREM-2 and CX3CR1. miR-101a inhibitor significantly decreased the number of Iba1^+^TREM-2^+^CX3CR1^+^ small, round cells, while miR-101a increased the number of these. *Bar* = 50 μm. **c**–**f** The numbers of cells positive for **c** Iba1, **d** CD11b, **e** TREM-2, and **f** CX3CR1 (*n* = 5). Data are presented as means ± S.D. *P < 0.05, **P < 0.01 by Mann-Whitney test

We next investigated the effect of miR-101a on the expression of microglial surface markers. Confocal microscopy revealed that although all cells were positive for CD11b, only SR cells expressed Iba1 (Fig. 3a). SR cells but not LF cells were positive for both TREM-2 and CX3CR1 (Fig. 3b). miR-101a inhibitor did not affect the number of CD11b^+^ or Iba1^+^ SR cells (Fig. 3c, d). In contrast, miR-101a inhibitor decreased the number of both TREM-2^+^ and CX3CR1^+^ cells, whereas miR-101a mimic increased these numbers (Fig. 3e, f). When compared to untreated cells, miR-101a inhibitor did not change the number of TREM-2^+^ and CX3CR1^+^ cells since control inhibitor significantly increased these cells (Fig. 3e, f). However, miR-101a mimic increased the number of TREM-2^+^ and CX3CR1^+^ cells even when compared to untreated cells (Fig. 3e, f). These data indicate that SR cells more closely resemble microglia than LF cells with respect to the expression of Iba1, TREM-2, and CX3CR1 and that miR-101a positively regulates the development of microglia-like cells.

### miR-101a is expressed in the murine CNS

**Fig. 4:**
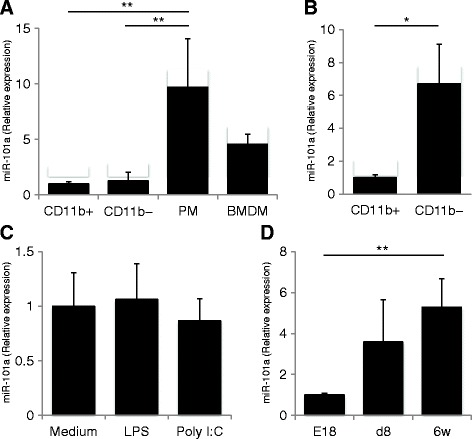
The expression level of miR-101a in vitro and in vivo. **a** Mixed glial cells were divided into CD11b^+^ cells or CD11b^−^ cells using CD11b-conjugated magnetic beads. The relative expression levels of miR-101a were analyzed by quantitative PCR and were compared with peritoneal macrophage (PM) and bone marrow-derived macrophages (BMDM). **b** CNS cells from C57BL/6J mice were divided into CD11b^+^ cells or CD11b^−^ cells with the same method. **c** The effects of lipopolysaccharide and poly I:C on the expression of miR-101a in MG6 immortalized microglial cells. **d** miR-101a expression in E18, postnatal d8, and 6 weeks brains. The expression levels of miR-101a were analyzed by quantitative PCR (*n* = 4) and were shown as relative expression to sno202. Data are presented as means ± S.D. *P < 0.05, **P < 0.01 by Student’s *t* test or ANOVA followed by Tukey’s post hoc test

We next measured the expression levels of miR-101a in vitro and in vivo. When mixed glial culture cells were divided into CD11b^+^ cells and CD11b^−^ populations using magnetic beads, CD11b^−^ cells expressed slightly higher levels of miR-101a than CD11b^+^ microglia (Fig. 4a). miR-101a was significantly more abundant in peritoneal macrophage than cultured microglia (Fig. 4a). Among adult brain cells, miR-101a was more highly expressed in the CD11b^−^ population compared to the CD11b^+^ population (Fig. 4b). Neither lipopolysaccharide (LPS) nor poly I:C stimulation altered the expression level of miR-101a (Fig. 4c). The expression level of miR-101a in adult brain was significantly higher than in fetal brain (Fig. 4d). These results indicate that miR-101a is relatively enriched in CD11b^−^ cells from adult brain.

### miR-101a modulates microglial proinflammatory cytokine expression

**Fig. 5:**
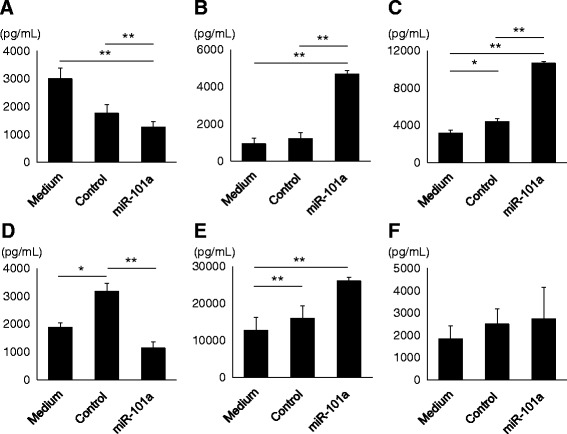
The effect of miR-101a on cytokine production. **a**, **d** IL-1β production from MG6 cells (**a**) or LN^−^ cell-astrocyte co-culture (**d**) after LPS plus ATP stimulation. **b**, **e** IL-6 production from MG6 cells (**b**) or LN^−^ cell-astrocyte co-culture (**e**) after LPS stimulation. **c**, **f** TNFα production from MG6 cells (**c**) or LN^−^ cell-astrocyte co-culture (**f**) after LPS stimulation. Levels of IL-1β, IL-6, and TNFα were measured by ELISA (*n* = 3). Data are presented as means ± S.D. *P < 0.05, **P < 0.01, ***P < 0.001 by Student’s *t* test or ANOVA followed by Tukey’s post hoc test

We investigated the effect of miR-101a on the character of microglia-like cells. Transfection of exogenous miRNA inhibitor or mimic did not affect cell viability of microglia cell line MG6 (Additional file 1: Figure S2). miR-101a treatment significantly decreased the production of IL-1β from MG6 cells compared to untreated or control-treated cells (Fig. 5a). In contrast, transfection of the miR-101a mimic significantly increased the production of IL-6 (Fig. 5b) and TNFα (Fig. 5c) from MG6 cells in response to LPS. Transfection of miR-101a mimic decreased the production of IL-1β from LN^−^ cell-astrocyte co-culture while the difference was not significant when compared to untreated cells because control mimic treatment increased IL-1β production (Fig. 5d). Transfection of miR-101a mimic increased IL-6 production (Fig. 5e) but did not alter the secretion of TNFα (Fig. 5f) from LN^−^ cell-astrocyte co-culture. These results indicate that miR-101a modulates expression of proinflammatory cytokines in microglia.

### miR-101a targets microglial Mkp-1

**Fig. 6:**
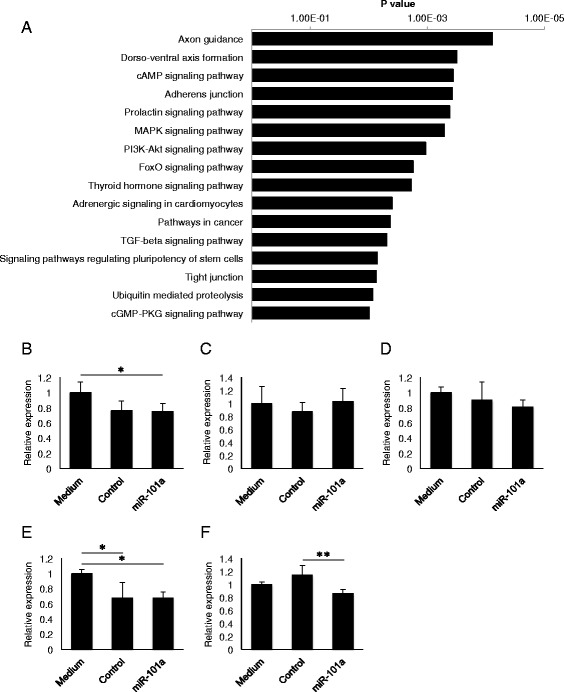
Target genes of miR-101a. **a** KEGG pathways enriched in miR-101a target genes. Target genes of miR-101a were predicted by TargetScan algorithm and pathways were analyzed by DAVID Functional Annotation Bioinformatics Microarray Analysis. **b-f** Quantification of mRNA expression of miR-101a target genes (FOS (**b**), NLK (**c**), SRF (**d**), Akt1 (**e**), and Mkp1 (**f**)) in MG6 cells treated with LPS (20 ng/mL, 24 h) by real-time PCR (*n* = 4). Beta-actin was used for normalization. Data are presented as means ± S.D. *P < 0.05, **P < 0.01 by ANOVA followed by Tukey’s post hoc test

Finally, we sought to identify target genes of miR-101a using TargetScan algorithm (TargetScanMouse 6.2, http://www.targetscan.org/mmu_61/). KEGG pathways enriched in miR-101a target genes (analyzed by DAVID; https://david.ncifcrf.gov/) included pathways in axon guiding, dorso-ventral axis formation, cAMP signaling, adherens junction, etc (Fig. 6a). Among them, MAPK signaling pathway is closely associated with the production of inflammatory cytokines (Additional file 2: Figure S3). We investigated the effect of miR-101a on the expression of several molecules involved in the MAPK pathway and identified MAPK phosphatase-1 (Mkp-1) as a target gene that was downregulated by miR-101a (Fig. 6b). Since Mkp-1 is known to suppress innate immune response, it was speculated that miR-101a regulate microglial inflammation by controlling Mkp-1 expression. In addition, TGF-beta signaling pathway may also be involved in the regulation of cytokine production.

## Discussion

Comparative microarray analyses of microglia have identified a unique transcriptome that is distinct from other macrophage cell types. This includes many neuron-associated transcripts such as synaptic molecules [15]. Microglial progenitors arise from the yolk sac and migrate to the fetal brain, where they develop under the influence of the CNS environment [7]. It is thus logical to speculate that some factor that is enriched in the brain facilitate microglia to acquire a distinct phenotype as a CNS-biased cell. miRNAs are a candidate factor for determining the specific phenotype of microglia.

In the present study, we identified miR-101a as a modifier of microglial morphology and function using a miRNA inhibitor library. It has been reported that primary macrophage is difficult to transfect siRNA or plasmid DNA due to reduced cell vitality and severely altered cell behavior [16]. This also hampers the study on the effect of miRNAs on microglia. Due to this limitation, we screened the effect of miRNAs using LN^−^ cell-astrocyte co-culture. As a result, we identified miR-101a as a modifier of the shape of LN^−^ cell-derived cells.

In these experiments, transfection of control mimic or inhibitor significantly changed the character of cells. In general, transfection of control mimic or inhibitor tended to increase the number of LN^−^ cell-derived cells. Although miR-101a inhibitor decreased the number of total, SR, TREM-2^+^ and CX3CR1^+^ cells, the differences were not significant when compared to untreated control (Figs. 2b, c and 3e, f). Nevertheless, miR-101a mimic undoubtedly increased the number of these cells and increased cytokine production form MG6 cells (Figs. 2b, c, 3e, f and 5a–c) compared to both medium and control mimic. Although the mechanism of this phenomenon is unknown, we speculated that the transfection of miRNA had non-specific immune activation of these cells. Various RNAi and miRNA reagents, which differ in length and structure, have been reported to cause non-sequence-specific immune responses [17]. The activation of the cellular sensors of foreign RNA or DNA may lead to the induction of type I interferon and cytokine release. This effect depends mostly on the reagent length, structure, chemical modification, and concentration rather than on its specific sequence [18]. In addition, it may also depend on the method of RNA delivery into the cells. However, control miRNA inhibitor that can cause such response would not be a good candidate for control when studying immune response of the cells. Thus, control miRNA and their delivery method should be carefully chosen to minimize non-specific effects. The RNAimmuno database (http://rnaimmuno.ibch.poznan.pl) provides information regarding the non-specific effects generated by RNA interference triggers and miRNA regulators [17].

miR-101a is downregulated in various types of cancer and functions as a tumor suppressor by repressing the polycomb group protein EZH2 [19]. miR-101a also inhibits tumor growth by regulating the cyclooxygenase-2 pathway [20]. miR-101a is enriched in the brain and regulates amyloid precursor protein expression in rat hippocampal neurons cultured in vitro [21]. However, the role of miR-101a in microglia was previously unknown.

We demonstrated that miR-101a affects microglia in two different ways. First, miR-101a promotes the development of uncommitted hematopoietic progenitors into myeloid cells that resemble microglia. Our previous study indicated that hematopoietic cells had the potential to develop into microglia [11]. Our model, in which bone marrow cells are co-cultured with astrocytes before differentiating into microglia-like cells, enables us to track the process by which non-microglial cells acquire phenotypes that are characteristic of microglia as well as identify factors that are essential for this process. In this model, miR-101a facilitates induction of TREM-2-positive microglia-like cells from bone marrow lineage-negative cells. The expression of TREM-2 in microglia is significantly higher than in other monocyte-macrophage lineage cells [13], indicating that miR-101a favored the commitment of LN^−^ cells to microglia. Nevertheless, it is unlikely that miR-101a plays a crucial role in the development of microglia in the fetal brain because this miRNA is not enriched in the fetal brain. Another possibility is that miR-101a may function to maintain the cellular character of microglia in the adult brain.

Second, miR-101a regulates proinflammatory cytokine expression in microglia. miR-101a increased the production of IL-6 and TNFα from microglia. In contrast, miR-101a decreased the production of IL-1β from LN^−^ cell-astrocyte co-culture. These results suggest that miR-101a regulates microglial inflammation through several diverse pathways. miRNAs exert their effects by targeting certain downstream molecules. We predicted several pathways enriched in the target genes of miR-101a, and they included MAPK and TGF-beta signaling pathways. With regard to MAPK signaling pathway, miR-101a is reported to enhance the transcription of inflammatory mediators by suppressing MKP-1 activity in RAW264.7 macrophages [22]. In the present study, miR-101 suppressed the expression of MKP-1 in LPS-treated MG6 cells. It is also reported that miR-101 mimics increased the production of TNFα [23]. These results suggest that miR-101 augment the production of both IL-6 and TNFα from microglia by inhibiting MKP-1.

## Conclusions

In conclusion, we have demonstrated that miR-101a, which is enriched in the brain, promotes the differentiation of bone marrow-derived microglia-like cells and cytokine production from microglia. These results shed new light on the molecular basis of microglial differentiation.

## Additional files

Additional file 1: Figure S1. The expression level of miR-101a in MG6 cells cultured with each miRNA was analyzed by quantitative real-time PCR. **Figure S2.** Neither miR-101a nor its inhibitor exhibited any influence on the viability of MG6 cells as measured by MTT assay. (PPTX 52 kb)

Additional file 2: Figure S3. Predicted miR-101a target genes included in MAPK signaling pathway. Asterisks indicate miR-101a targets. (TIF 101 kb)

## Abbreviations

Ab: Antibody
B6: C57BL/6J
CNS: Central nervous system
GFP: Green fluorescent protein
LF cells: Large, flat cells
LN^−^cells: Lineage-negative cells
miRNA: MicroRNA
Mkp-1: MAPK phosphatase-1
SR cells: Small, round cells
TREM-2: Triggering receptor expressed on myeloid cells (TREM)-2

## References

[1] Ha M, Kim VN (2014). Regulation of microRNA biogenesis. Nat Rev Mol Cell Biol.

[2] Kosik KS (2006). The neuronal microRNA system. Nat Rev Neurosci.

[3] Ponomarev ED, Veremeyko T, Weiner HL (2013). MicroRNAs are universal regulators of differentiation, activation, and polarization of microglia and macrophages in normal and diseased CNS. Glia.

[4] Ponomarev ED, Veremeyko T, Barteneva N, Krichevsky AM, Weiner HL (2011). MicroRNA-124 promotes microglia quiescence and suppresses EAE by deactivating macrophages via the C/EBP-alpha-PU.1 pathway. Nat Med.

[5] Moore CS, Rao VT, Durafourt BA, Bedell BJ, Ludwin SK, Bar-Or A (2013). miR-155 as a multiple sclerosis-relevant regulator of myeloid cell polarization. Ann Neurol.

[6] Yao H, Ma R, Yang L, Hu G, Chen X, Duan M (2014). MiR-9 promotes microglial activation by targeting MCPIP1. Nat Commun.

[7] Ginhoux F, Greter M, Leboeuf M, Nandi S, See P, Gokhan S (2010). Fate mapping analysis reveals that adult microglia derive from primitive macrophages. Science.

[8] Gomez Perdiguero E, Klapproth K, Schulz C, Busch K, Azzoni E, Crozet L (2015). Tissue-resident macrophages originate from yolk-sac-derived erythro-myeloid progenitors. Nature.

[9] Wang Y, Szretter KJ, Vermi W, Gilfillan S, Rossini C, Cella M (2012). IL-34 is a tissue-restricted ligand of CSF1R required for the development of Langerhans cells and microglia. Nat Immunol.

[10] Priller J, Flugel A, Wehner T, Boentert M, Haas CA, Prinz M (2001). Targeting gene-modified hematopoietic cells to the central nervous system: use of green fluorescent protein uncovers microglial engraftment. Nat Med.

[11] Noto D, Takahashi K, Miyake S, Yamada M (2010). In vitro differentiation of lineage-negative bone marrow cells into microglia-like cells. Eur J Neurosci.

[12] Noto D, Sakuma H, Takahashi K, Saika R, Saga R, Yamada M (2014). Development of a culture system to induce microglia-like cells from haematopoietic cells. Neuropathol Appl Neurobiol.

[13] Colonna M (2003). TREMs in the immune system and beyond. Nat Rev Immunol.

[14] Takenouchi T, Ogihara K, Sato M, Kitani H (2005). Inhibitory effects of U73122 and U73343 on Ca2+ influx and pore formation induced by the activation of P2X7 nucleotide receptors in mouse microglial cell line. Biochim Biophys Acta.

[15] Beutner C, Linnartz-Gerlach B, Schmidt SV, Beyer M, Mallmann MR, Staratschek-Jox A (2013). Unique transcriptome signature of mouse microglia. Glia.

[16] Maess MB, Wittig B, Lorkowski S. Highly efficient transfection of human THP-1 macrophages by nucleofection. J Vis Exp. 2014;e51960. 10.3791/51960 PMC4828023 25226503

[17] Olejniczak M, Galka-Marciniak P, Polak K, Fligier A, Krzyzosiak WJ (2012). RNAimmuno: a database of the nonspecific immunological effects of RNA interference and microRNA reagents. RNA.

[18] Olejniczak M, Galka P, Krzyzosiak WJ (2010). Sequence-non-specific effects of RNA interference triggers and microRNA regulators. Nucleic Acids Res.

[19] Varambally S, Cao Q, Mani RS, Shankar S, Wang X, Ateeq B (2008). Genomic loss of microRNA-101 leads to overexpression of histone methyltransferase EZH2 in cancer. Science.

[20] Strillacci A, Griffoni C, Sansone P, Paterini P, Piazzi G, Lazzarini G (2009). MiR-101 downregulation is involved in cyclooxygenase-2 overexpression in human colon cancer cells. Exp Cell Res.

[21] Vilardo E, Barbato C, Ciotti M, Cogoni C, Ruberti F (2010). MicroRNA-101 regulates amyloid precursor protein expression in hippocampal neurons. J Biol Chem.

[22] Gao Y, Liu F, Fang L, Cai R, Zong C, Qi Y (2014). Genkwanin inhibits proinflammatory mediators mainly through the regulation of miR-101/MKP-1/MAPK pathway in LPS-activated macrophages. PLoS One.

[23] Zhu QY, Liu Q, Chen JX, Lan K, Ge BX (2010). MicroRNA-101 targets MAPK phosphatase-1 to regulate the activation of MAPKs in macrophages. J Immunol.

