# Supplementary material for: MicroRNA-101a regulates microglial morphology and inflammation
Source: J Neuroinflammation. 2017 May 30;14:109. doi: 10.1186/s12974-017-0884-8 (PMC5450088; doi:10.1186/s12974-017-0884-8)
Supplement: Supplementary file 1 — The expression level of miR-101a in MG6 cells cultured with each miRNA was analyzed by quantitative real-time PCR. Figure S2. Neither miR-101a nor its inhibitor exhibited any influence on the viability of MG6 cells as measured by MTT assay. (PPTX 52 kb) [file 12974_2017_884_MOESM1_ESM.pptx]

## Slide 1
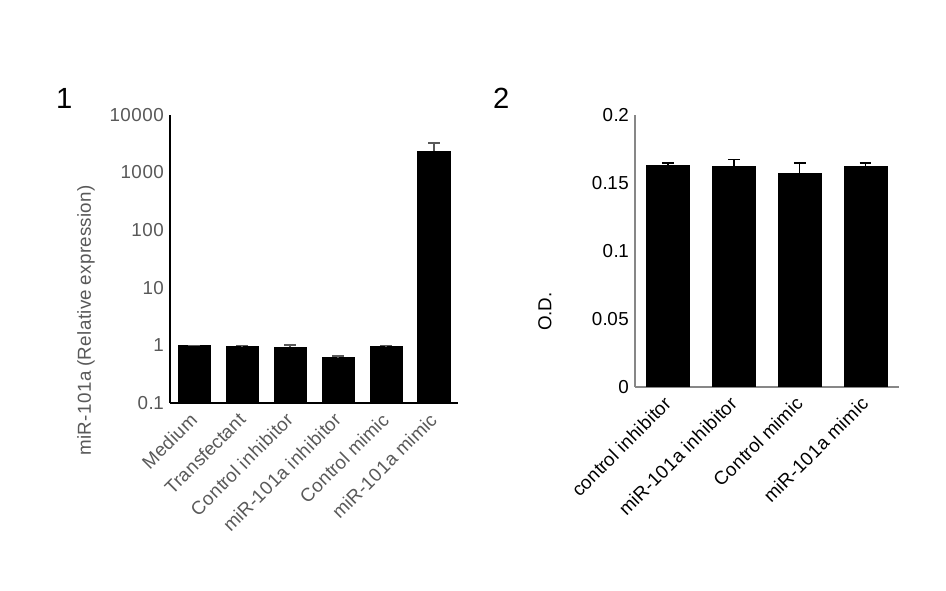

1
2
### Chart
| Category | AVG |
|---|---|
| Medium | 1.0 |
| Transfectant | 0.954836214400449 |
| Control inhibitor | 0.930414477045448 |
| miR-101a inhibitor | 0.615807532550318 |
| Control mimic | 0.946272362801509 |
| miR-101a mimic | 2377.784036164353 |
### Chart
| Category | Cell viavility |
|---|---|
| control inhibitor | 0.163420666666667 |
| miR-101a inhibitor | 0.162394666666667 |
| Control mimic | 0.156974 |
| miR-101a mimic | 0.162203333333333 |
