# Supplementary figures and images for: MicroRNA-101a regulates microglial morphology and inflammation
Source: J Neuroinflammation. 2017 May 30;14:109. doi: 10.1186/s12974-017-0884-8 (PMC5450088; doi:10.1186/s12974-017-0884-8)

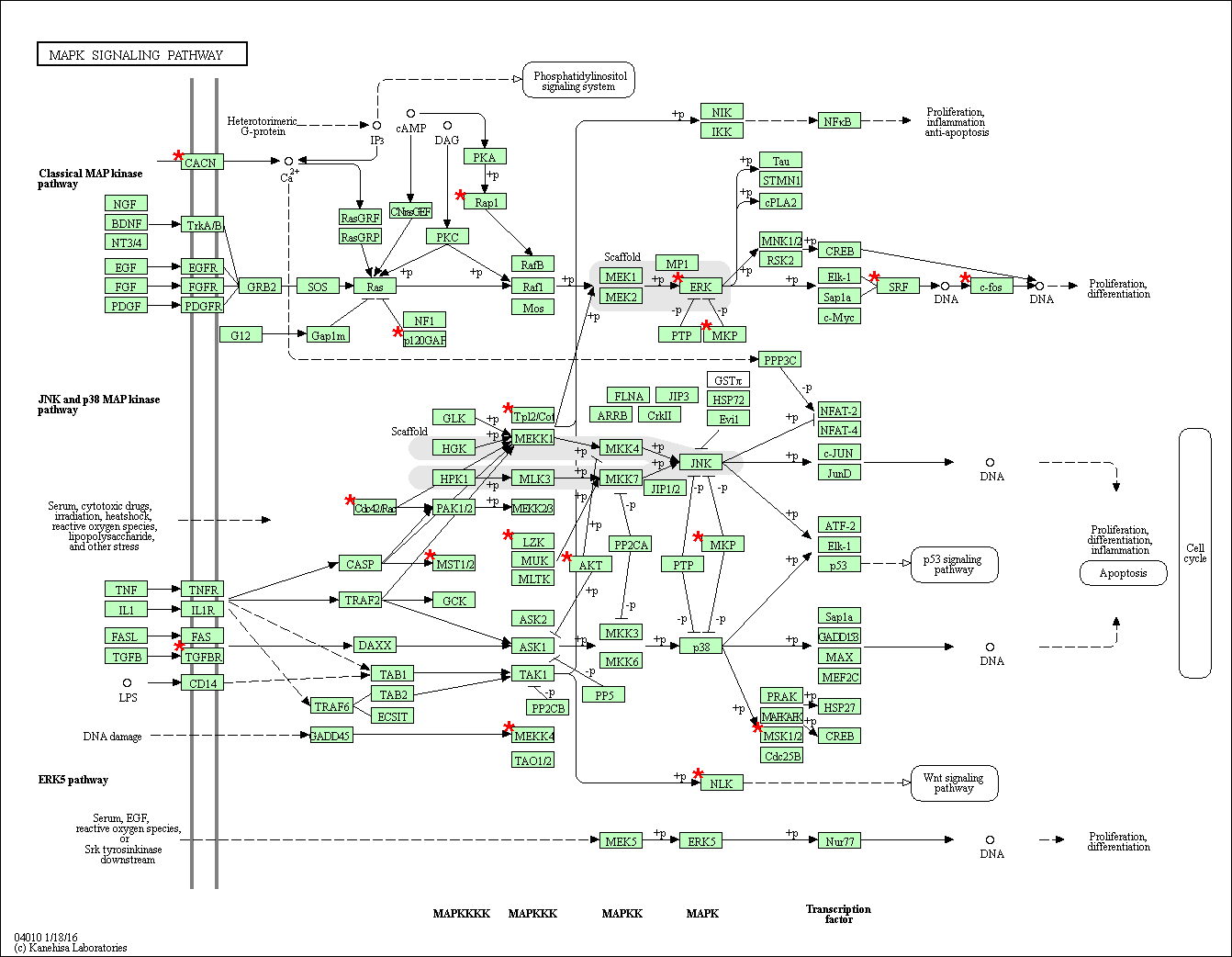

Supplement: Supplementary file 2 — Predicted miR-101a target genes included in MAPK signaling pathway. Asterisks indicate miR-101a targets. (TIF 101 kb) [file 12974_2017_884_MOESM2_ESM.tif]
